# Infants and young children feeding practice and associated factors among HIV positive mothers of children 0–23 months in health centers of Gulele sub-city, Addis Ababa, Ethiopia

**DOI:** 10.1186/s13104-019-4729-7

**Published:** 2019-10-21

**Authors:** Samuel Negash, Firehiwot Mesfin, Gudina Egata

**Affiliations:** 1 Department of Public Health, College of Health Sciences, Mizan-Tepi University, Mizan Aman, Ethiopia; 2 College of Health and Medical Science, School of Public Health, Haramaya University, Dire Dawa, Ethiopia

**Keywords:** Ethiopia, Feeding practice, HIV, Infants

## Abstract

**Objective:**

A health facility based cross sectional study design was conducted among 358 randomly selected HIV positive mothers attending at four health centers from February 1 to 28, 2018. Magnitude of HIV positive mothers’ child feeding practice and associated factors was assessed according to WHO recommendation. Data were collected using structured pretested questionnaire and entered into EPI data version 3.1 and exported to SPSS version 20 computer software for analysis.

**Result:**

The magnitude of recommended way of infant feeding practice among HIV positive mothers attending public health centers in Gulele sub-city is 37.4%, 95% CI (32.26–42.67). Statistically significant correlates of HIV exposed infant feeding practice of mothers in this study were knowledge of mother on HIV exposed infant feeding practice (AOR = 1.80 (95% CI 1.04–3.01)), head of family being father (AOR = 0.17 (95% CI 0.03–0.87)), having family (relatives) support (AOR = 2.05 (95% CI 1.00–4.18)) and information on HIV exposed infant feeding, practice (AOR = 1.77 (95% CI 1.07–2.93)).

## Introduction

Infant and young child feeding is a cornerstone of care for childhood development. The first 2 years of life provides a critical window of opportunity to ensure children appropriate growth and development from generation to generation through optimal feeding [1].

Globally, the numbers of people living with HIV under Anti-Retro Viral Therapy (ART) were about 17 million; each year 2.1 million cases on average are added stagnantly to sum up to 39.8 million by the end of 2015. But thanks to Prevention of Mother to Child Transmission (PMTCT) efforts, 1.6 million new pediatric infections were globally prevented from 2000 onwards [2].

According to the World Health Organization (WHO)’s revised guideline, Mothers known to be HIV-infected and whose infants are HIV uninfected or of unknown HIV status should exclusively breastfeed their infants for the first 6 months of life, introducing an appropriate complementary foods thereafter and continue breastfeeding for 12 months [2].

In Ethiopia 74% of all women have knowledge of HIV transmission through breast milk and 58% of infants under 6 months were on Exclusive Breast Feeding (EBF), 17% fed with plain water and 5% were not breast fed [3]. Federal Ministry of Health (FMOH) adopted a guideline from WHO 2010/2013 report on Option B+ regimen with a goal to provide extended prevention of new pediatric infection [4].

Inadequate counseling, follow up and family support to HIV positive mothers as well as shortage of training to counselors and related health professionals on timely and important nutritional messages to follow the recommended way of HIV exposed infant and young children feeding practice were some of the main obstacles being difficulties towards minimizing the transmission of HIV [5].

Finally, there were limited numbers of previous studies conducted in the study area on this issue. Therefore, knowing the status of feeding in the critical stages of infancy under the risk of HIV has a lot to do with improving the quality of life in this segment of population. Hence, this study was conducted to assess the magnitude and associated factors of HIV positive mothers feeding practice to their infant and young children according to WHO recommendation which is defined as ‘using EBF or Exclusive Replacement Feeding (ERF) up to 6 months followed by complementary feeding with breast feeding continued at least for 12 months’.

## Main text

### Methods

#### Study setting

The study was conducted in Gulele sub- city which is one of the ten sub-cities of Capital city, Addis Ababa with a total population of 267,624 dwelling in ten weredas of which 52% comprises female population according to 2011 city government’s report.

#### Sample size and sampling procedure

Sample size for the first objective was calculated using single population proportion formula with the following assumptions—the prevalence of infant feeding practice taken from previous study in Addis Ababa town which was 69.7% with confidence level of 95% marginal error of 5% and 10% for non-response rate [6]. Therefore, the sample size of the first objective was taken and the final sample size was 358.

#### Data collection and analysis

Data were collected by recruiting HIV positive mothers who came for their monthly appointment at selected health centers for Anti-natal and Anti-retro-viral therapy monthly follow up after confidentiality issues like oral consent were addressed by using their health care givers as interviewer after 2 days training was offered prior to data collection for data collectors.

The outcome of the study ‘Feeding practice’ of HIV positive mothers for their HIV exposed child was dichotomized into Recommended feeding practice and Not recommended feeding practice as per WHO’s set criteria for Exclusive breast feeding (EBF), Exclusive replacement feeding (ERF) and Mixed feeding (MF) considered EBF or ERF as recommended by preference and MF as not recommended in the age groups up to 6 months and stays up to 12 months of age.

During the interview, from socio demographic characteristics, their educational status was assessed as ‘not formally educated; or ‘formally educated in primary, secondary schools and above. Other independent variables like Age of the mother and child, marital status, Disclosure of HIV status and related questions were addressed Whereas, when Family support was considered, if there was any body other than her spouse, from family or relatives aspect who supported the mother in deciding what and how to feed her child.

The knowledge of mother on the ways of feeding practice and her information source to use appropriate feeding practice were also assessed by standard questions prepared separately for both factors. In this case the ultimate goal of the study is to measure or identify the general knowledge and information on recommended feeding practice irrespective of her child’s experience for instance, if she had child under 6 month, she was asked not only about her practice but also what she has planned next and up to what extent was her preparedness and information on how to feed her child.

The collected data were entered into EPI data version 3.1 and exported to SPSS version 20 computer software for analysis. Descriptive statistics was used to describe the study variables. Bivariable logistic regression was used to see the association between each dependent variables and the outcome variable.

Continuous variables like age, family size and monthly income were first transformed into categorical variable and those with p-value less than or equal to 0.25 were analyzed into multivariable logistic regression, level of statistical significance was declared at p-value less than or equal to 0.05. Model fitness was checked by using Hosmer and Lemshow goodness of fit and multicollinearity between dependent and independent variables was checked to have lesser variance inflation factor and by using standard error with lower values less than 2 that indicate more precise estimates and finally significantly associated variables with the outcome variable were identified.

### Result

#### Socio-demographic characteristics of the study participants

**Fig. 1:**
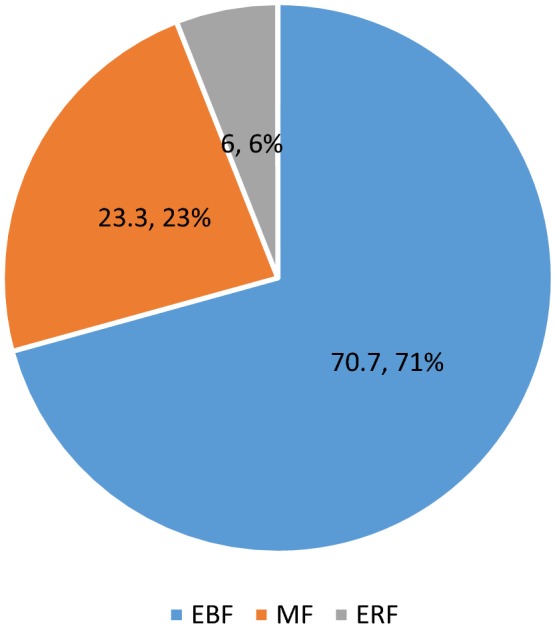
Percentage of infant feeding practice of HIV positive mothers of under 6 month infants in selected public health centers of Gulele sub-city, 2018

From a total of 358 HIV positive mothers recruited, 348 of them were participated in the study making the response rate 97.2%. The mean age of mothers was 29.14 (± SD 5.50) years and for infants 8.47 (± SD 6.09) months. Majority of the mothers, 296 (85.1%) were married with age 40 (11.5%) having not attended formal education. The mean family size was 3.97 with standard deviation of 1.155. Concerning total family monthly income, most of them 218 (62.6%) had an income between 1500 and 5000 ETB with mean monthly income of 3964.97 Ethiopian Birr (ETB) (Table 1, Fig. 1).

**Table 1:** Socio demographic characteristics of HIV positive mothers of HIV exposed infant and young children 0–23 months of age attending public health centers in Gulele sub-city, 2018 (n = 348)

Variable	Frequency	Percent (%)
Age of mothers		
16–19	5	1.4
20–29	182	52.3
30–39	146	42.0
40–49	15	4.3
Age group of the children		
≤ 5 months	162	46.6
6–11 months	85	24.4
12–17 months	67	19.2
18–24 months	34	9.8
Marital status		
Married	296	85.1
Divorced	24	6.9
Single	14	4.0
Widowed	14	4.0
Mothers educational level		
No formal education	40	11.5
Formal education	308	88.5
Occupation of mothers		
Housewife	126	36.2
Student	40	11.5
Private employee	84	24.1
Government employee	60	17.2
Merchant	38	10.9
Husband’s occupation		
Unemployed	22	6.3
Private employee	111	31.9
Government employee	113	32.5
Merchant	67	19.3
Household monthly income		
≤ 1500	50	41.9
1501–5000	218	62.46
< 5001	80	23.0
Head of family		
Mother	60	17.2
Father	270	77.6
Parents	18	5.2
Family support		
Family	133	38.2
Neighbors	11	3.2
Relatives	67	19.3
NGOs	32	9.2
Nobody	105	30.2

#### Factors affecting HIV exposed infant and young child feeding practice of HIV positive mothers

In this study, the magnitude of WHO recommended feeding practice is 37.4% CI (32.26–42.67) in bivariable logistic regression analysis, knowledge of mothers on infant feeding [COR = 1.87 (95% CI 1.16–3.03)] family support from relatives [COR 1.99 (95% CI 1.04–3.78)], head of family [COR 0.17 (95% CI 0.04–0.79)] and information of mothers on infant feeding [COR 1.71 (95% CI 1.10–2.66)] were found to have association (p-value < 0.25) with the recommended way of infant feeding practice among all variables entered. Whereas, 99 (40%) of mothers disclosed their HIV status to their partner and 73 (29.67%) to their parents.

In multivariate analysis, knowledge of mother on HIV exposed infant feeding [AOR = 1.80 (95% CI 1.04–3.01)], head of family being father AOR = 0.17 (95% CI 0.03–0.87), family support AOR = 2.05 (95% CI 1.00–4.18) and information on HIV exposed infant feeding AOR = 1.77 (95% CI 1.07–2.93) were found to be independently associated (p-value < 0.05) with recommended way of infant feeding practice and respectively (Table 2).

**Table 2:** Bivariable and multivariable logistic regression analysis showing relation between the outcome selected and associated variables of HIV positive mothers feeding practice

Variables	Feeding practice		COR [95% CI]	AOR [95% CI]
	RFP (%)	NRFP (%)		
Mothers’ educational status				
Not formally educated	15 (37.5)	25 (62.5)	0.99 (0.50–1.94)	
Formally educated	115 (37.3)	193 (62.7)	1	
Family monthly income				
1501–5000	86 (39.4)	132 (60.6)	0.48 (0.24–0.98)	
> 5001	32 (40.0)	48 (60.0)	0.4 (0.21–1.04)	
≤ 1500	12 (24.0)	38 (76.0)	1	
ANC follow up				
Yes	129 (37.89)	212 (62.2)	0.27 (0.23–2.30)	
No	1 (14.3)	6 (85.7)		
On ART^				
Yes	124 (37.1)	210 (62.9)	1.27 (0.43–3.74)	
No	6 (42.9)	8 (57.1)	1	
Knowledge of mothers				
Adequate	97 (42.2)	133 (57.8)	1.87 (1.16–3.03)	1.80 (1.04–3.01)*
Inadequate	33 (28.0)	85 (72.0)	1	1
Family support on infant feeding				
Family	41 (30.8)	92 (69.2)	2.04 (1.19–3.47)	1.35 (0.73–2.50)
Neighbors	4 (36.4)	7 (63.6)	1.59 (0.43–5.76)	1.34 (0.32–5.51)
Relatives	21 (31.3)	46 (68.7)	1.99 (1.04–3.78)	2.05 (1.00–4.18)*
NGOs	14 (43.8)	18 (56.2)	1.16 (0.52–2.58)	0.75 (0.30–1.87)
Nobody	50 (47.6)	55 (52.4)	1	1
Information of mother on feeding practice				
Adequate	77 (87.4)	100 (56.5)	1.71 (1.10–2.66)	1.77 (1.07–2.93)*
Inadequate	53 (31.0)	118 (69.0)	1	1
Head of family				
Mother	17 (28.3)	43 (71.7)	0.3 [0.66–1.52]	0.31 [0.05–1.68]
Father	111 (41.1)	159 (58.9)	0.17 [0.04–0.79]	0.17 [0.03–0.87]*
Parents	2 (11.1)	169 (88.9)	1	1
Disclosure status				
Yes	88 (35.8)	158 (64.2)	1.25 [0.78–2.01]	
No	42 (43.5)	60 (58.8)	1	

*RFP* recommended feeding practice, *NRFP* not recommended feeding practice

* Statistically significant (p-value < 0.05); ^ variables with p-value > 0.25, hence not included in the multivariable model

### Discussion

This study has tried to focus on infant feeding practice of HIV positive mothers of infants 0–23 months in Gulele sub-city selected public health centers. In this study, the magnitude of WHO recommended feeding practice is 37.4% CI (32.26–42.67) which is much less than the study in Coastal Tanzania reported as 77% prevalence of EBF and 50% for prevalence of complementary feeding [7] that the difference might be arisen due to the variation in feeding culture and level of mother’s knowledge on feeding between two countries. Particularly, in this study magnitude of EBF was 70.7% which is in line with the national target set in Ethiopia (70%) by 2020 [5].

Family support which is expressed in relatives is one of the factors which were significantly associated with recommended way of feeding practice, only 30.4% of HIV positive mothers in this study have nobody to support despite most of the study participants had husband support, related study in Gondar reported lesser proportion (9.6%) of husband opposition [8]. While according to this study, mothers who had family support from relatives were 2.05 times to practice recommended way of feeding practice which is consistent with the study in Guraghe zone that revealed mothers with family support had practiced EBF 2 times more than those who were not supported [9]. This shows support to HIV positive mothers from the family and relatives has great impact to have more appropriate way of feeding by sharing knowledge and information each other than being idle.

Knowledge of mothers on breastfeeding is very important for the mother to make better decision on infant feeding options. It is the basic ground for practice arisen from having an information. This study showed that HIV positive mothers who had adequate knowledge on infant feeding were 1.8 times more likely to have recommended way of infant feeding practice than those who had inadequate knowledge which is supported by a study conducted in Guraghe zone, that showed 1.9 times more likely to have recommended way that can be justified by similarity in age and health care service they might got throughout the country [8, 10]. This finding is in line with similar researches conducted in Addis Ababa and Mekele due to the fact that, in most low income countries female headed families face economic problems that hinder them to have appropriate feeding choice [11]. Moreover, families with nobody to take care or supported by grandparent might fail in setting necessary economical support that makes the difference from which both parents take over [12].

## Conclusion

The present study identified that the magnitude of infant feeding practice was very low. Similar with other studies conducted in Ethiopia, proportion of complementary practice was lowers than EBF practice. Factors which are influencing infant feeding practice were found to be mother’s knowledge on HIV exposed infants feeding practice, information on feeding practice, head of family and family support.

According to the study Mixed feeding is one of the challenges that hinder attaining WHO recommended way of feeding practice in the study area. It was also observed that, only some mothers experienced Exclusive Replacement Feeding.

## Limitation of the study

Since there is need of information about early feeding practice of mothers about their child possible limitations were encountered like recall bias and social desirability bias. Due to the nature of the study participants causality issues in related to their health status and other social aspects were also expected compared to studies that were conducted on general population.

## Data Availability

Data for the conclusions of this study are included in the article. The data set is handled by corresponding author and can be provided upon request.

## Abbreviations

AFASS: acceptable feasible affordable sustainable and safe
AIDS: acquired immunity deficiency syndrome
ANC: anti natal care
AOR: adjusted odds ratio
ART: anti retro viral therapy
CI: confidence interval
CS: caesarian section
DDS: dietary diversity score
EBF: exclusive breast feeding
EDHS: Ethiopian Demographic Health Survey
MF: mixed feeding
IYCF: infant and young child feeding
NEC: nutrition education communication
NNP2: national nutrition program two
PMTCT: prevention of mother to child transmission
RFP: recommended feeding practice
SVD: spontaneous vertex delivery
UNICEF: United Nations International Child Education Fund
WHO: World Health Organization
